# The Impact of Sub-maximal Exercise on Neuropathic Pain, Inflammation, and Affect Among Adults With Spinal Cord Injury: A Pilot Study

**DOI:** 10.3389/fresc.2021.700780

**Published:** 2021-10-26

**Authors:** Kendra R. Todd, Jan W. Van Der Scheer, Jeremy J. Walsh, Garett S. Jackson, Gabriel U. Dix, Jonathan Peter Little, John L. K. Kramer, Kathleen A. Martin Ginis

**Affiliations:** ^1^Department of Kinesiology, University of British Columbia, Kelowna, BC, Canada; ^2^International Collaboration on Repair Discoveries, Vancouver Coastal Health Research Institute, Vancouver, BC, Canada; ^3^The Healthcare Improvement Studies Institute, University of Cambridge, Cambridge, United Kingdom; ^4^Department of Kinesiology, McMaster University, Hamilton, ON, Canada; ^5^Department of Anesthesiology, Pharmacology, and Therapeutics, University of British Columbia, Vancouver, BC, Canada; ^6^Department of Medicine, Division of Physical Medicine and Rehabilitation, University of British Columbia, Vancouver, BC, Canada; ^7^Centre for Chronic Disease Prevention and Management, University of British Columbia, Kelowna, BC, Canada

**Keywords:** neuropathic pain, exercise, disability, affect, inflammation

## Abstract

**Introduction:** Persons with spinal cord injury (SCI) often report high levels of neuropathic pain (NP) and poor well-being, which may result from increased inflammation. This study examined the impact of sub-maximal aerobic exercise on NP, inflammation and psychological affect among adults with SCI.

**Methods:** Eight active adults with tetraplegia (*n*-4, AIS A-C) and paraplegia (*n* = 4, AIS A-C) performed 30-min of arm-crank aerobic exercise and reported their ratings of perceived exertion (RPE) each minute. Measures of NP, affect, and inflammatory cytokines (IL-6, IL-10, IL-1ra, TNF-α) were taken pre-(T_0_), immediately post-(T_1_), and 90-min post-exercise (T_2_).

**Results:** NP decreased between T_0_ and T_1_ for tetraplegics (−60%, *d* = 0.47; CI = −0.32, 2.02) and paraplegics (−16%, *d* = 0.15; CI = −0.30, 0.90). Correlations between change in cytokines and change in NP were medium-to large for tetraplegics (*rs* ranged from −0.820 to 0.965) and paraplegics (*rs* ranged from −0.598 to 0.833). However, the pattern of correlations between change in cytokines and affect was inconsistent between groups. Lower baseline levels of IL-1ra predicted greater decreases in NP immediately post-exercise (*r* = 0.83, *p* = 0.01).

**Conclusion:** Sub-maximal exercise can positively impact NP for some persons with SCI. Further experimental research should identify the optimal exercise intensity to reduce NP for persons with SCI, in addition to understanding biomarkers which may predict changes in NP.

**Clinical Trial Registration:** www.ClinicalTrials.gov, identifier NCT03955523.

## Introduction

Neuropathic pain is caused by a lesion or disease of the somatosensory nervous system (1) and commonly manifests as allodynia (pain resulting from a non-noxious stimulus) and hyperalgesia [heightened response from a noxious stimulus; (2)]. Approximately 75% of persons with spinal cord injury (SCI) experience neuropathic pain (3), with many reporting pain to be more debilitating than the injury itself. Individuals with SCI who experience greater pain also report more negative affect [instantaneous displeasure that lacks cognitive appraisal; (4)] and susceptibility to developing mood disorders, such as depression or anxiety (5).

Current treatment options for neuropathic pain are primarily pharmaceutical; however, in addition to eliciting debilitating side effects, pharmaceuticals result in just 50% pain reduction for only 30% of individuals with SCI-related chronic neuropathic pain (6). Consequently, many individuals who experience neuropathic pain prefer non-pharmaceutical alternatives (7). In a survey of 90 adults with SCI, 79% of those who reported on their preference for neuropathic pain management, preferred non-pharmaceutical treatments (8). Together, these data demonstrate the need to identify alternative treatment options to treat neuropathic pain among individuals with SCI.

One potential treatment to alleviate SCI neuropathic pain, improve affect, and potentially reduce risk for mood disorders (9, 10) is exercise. Exercise has been recognized as having positive effects on neuropathic pain among persons with SCI, despite the limited quantity and quality of evidence (11). However, evidence is emerging which indicates that exercise induced hypoalgesia may exist for persons with SCI. For example, in a case series, participants with SCI reported decreased neuropathic pain sensations following at least one of two bouts of self-selected, community-based exercise performed within a single week (9). The neuropathic pain-modulating benefits of acute exercise appear to persist with exercise training, as 10 weeks of aerobic exercise training led to decreased neuropathic pain for adults with SCI upon completion of the intervention (12). Various biological mechanisms underlying exercise induced hypoalgesia have been explored among able-bodied individuals, with evidence suggesting an interplay between the opioid, endocannabinoid, serotonergic and immune systems (13–15). However, the explanations for why exercise modulates neuropathic pain among persons with SCI are currently unknown.

The mechanisms underpinning neuropathic pain development are not well-understood. Various hypotheses exist in attempts to explain neuropathic pain development including structural damage within the central nervous system, and somatosensory cortex reorganization (16). However, the nociceptive environment also impacts the presence and severity of neuropathic pain (17). Pro-inflammatory cytokines such as interleukin-6 (IL-6), and tumor necrosis factor-alpha (TNF-α) have been shown to induce hyperalgesia, thereby increasing levels of neuropathic pain (18). In contrast, a reduction in these pro-inflammatory cytokines has been shown to prompt analgesic effects (17, 18). This relationship has also been demonstrated among persons with SCI using a dietary intervention. Relative to a control group, participants who consumed an anti-inflammatory diet for 12 weeks experienced changes in sensory neuropathic pain as a function of the change in pro-inflammatory cytokines (19). It is not known whether exercise can elicit the same effects in people with SCI.

In persons without SCI, research consistently shows that exercise can lead to increased acute levels of circulating IL-6, and subsequent rises in plasma concentrations of anti-inflammatory cytokines IL-1 receptor antagonist (IL-1ra), IL-10, and soluble TNF receptors (20, 21). However, exercise needs to be performed at a minimum intensity (>50% VO_2_max) or duration (>30 min) for the anti-inflammatory response (21), analgesic effects (22) and improvements in affect (23) to occur. For persons with SCI above the 6th thoracic vertebrae (T6), the loss of somatic and autonomic control frequently results in a blunted cardiovascular response to exercise (24). Additionally, research evidence is inconclusive regarding the role and extent that the sympathetic nervous system plays in exercise-related changes in inflammation (25, 26) and subsequent analgesic effects. Although exercise can induce analgesia through upregulation of anti-inflammatory cytokines and reduced microglial activation in the central nervous system (27), it is unclear whether persons with SCI above T6 experience an attenuated analgesic response to exercise. Taken together, it is important to examine if any biomarkers can be used to predict who may experience exercise-related improvements in neuropathic pain following SCI.

The debilitating nature of neuropathic pain, coupled with the bidirectional relationship between pain and affect, highlight the need for understanding neuropathic pain as having both physiological and psychosocial contributors. Therefore, the primary purpose of this study was to test the effect of a single bout of sub-maximal aerobic exercise on inflammatory cytokines, neuropathic pain, and affect among individuals with SCI. Additionally, an exploratory aim of this study was to assess the relationship between baseline levels of cytokines and changes in neuropathic pain from pre- to post-exercise. Sub-maximal aerobic exercise was implemented in order to align with exercise guideline recommendations for people with SCI (28). It was hypothesized that a bout of sub-maximal aerobic exercise would lead to an acute increase in circulating levels of IL-6 and anti-inflammatory cytokines, decreased neuropathic pain, and improved affect from pre- to post-exercise among persons with SCI. Additionally, it was hypothesized that decreased neuropathic pain would be correlated with increased anti-inflammatory cytokines and improved affect.

## Methods

### Participants

To participate in this study, individuals were required to: (1) have incurred an SCI > 12 months ago with an injury at the third cervical level or below (as long as diaphragmatic control and arm functioning allowed upper-body exercise); (2) experience chronic below-level of SCI neuropathic pain (>3 months) (at-level of SCI pain was excluded to minimize the risk of pain misidentification). Chronic neuropathic pain was an inclusion criterion given that neuropathic pain typically persists >3 months for persons with SCI, and to ensure that pain did not spontaneously resolve between testing sessions; (3) individuals were required to have the ability to read/write in English; and (4) routinely achieve at least the lowest level of the SCI exercise guidelines consisting of 20 min of moderate-to-vigorous intensity aerobic activity two times per week, and strength training two times per week, consisting of three sets of 8–10 repetitions of each exercise for each major muscle group (28). The latter criterion was instated because acute exercise participation by chronically inactive individuals may induce pain “flare-ups” (29) and impact inflammatory profiles.

Participants were recruited January–March, 2019 through advertisements emailed from community organizations from across British Columbia, and through emails directed toward individuals who had previously expressed interest in participating in SCI-studies. After screening, 10 active individuals with SCI of >1-year duration participated in this study. Ten individuals were the desired sample size based on previous case series data (9). This study carried the approval of the UBC Clinical Research Ethics Board (CREB; H18-03191), whereby all experiments were performed in accordance with CREB guidelines and regulations. Participants provided written, informed consent prior to enrolling in this study.

### Procedural Overview

This study is a secondary analysis of case series data (clinicaltrials.gov registered: NCT03955523; 20/05/2019).

#### Measures

##### Inflammatory Cytokines

Blood samples were drawn by a trained phlebotomist from participants' most accessible antecubital vein. Samples were collected in the same clinical laboratory room as the exercise bout. All biosafety hazard protocols were followed, and JL's laboratory held a UBC-approved biosafety permit for this research space. Samples were placed in EDTA tubes and centrifuged at 2,000 g for 15 min at 4°C (Eppendorf, Hamburg, Germany), followed by a subsequent centrifuge at 10,000 g for 10 min at 4°C to remove platelets. The resultant supernatant was subsequently aliquoted and stored at −80°C. IL-6, IL-1ra and TNF-α were analyzed and quantified using the U-PLEX metabolic group 1 assay kits (K151ACL-1, LOT 289109, Mesoscale, Maryland, USA) according to manufacturers' instructions, and read using an MSD QuickPlex SQ120 plate reader (Mesoscale, Maryland, USA). All samples were analyzed in duplicate. The plate-specific intra-assay coefficients of variation (CV) were as follows: IL-10 = 10.4%; IL-1ra = 5.91%; IL-6 = 13.73%; TNF-α = 17.84%. The combined CV was 11.97%.

##### Neuropathic Pain Scale

Participants' neuropathic pain was measured using a modified version of the Neuropathic Pain Scale [NPS; (30)]. This 10-item scale (0 = nothing at all, 10 = most intense sensation imaginable) measures pain sensations common to neuropathic pain (e.g., “burning,” “dull,” “deep”) in addition to measuring general pain qualities (i.e., “intensity” and “unpleasantness”). For each item on the NPS, participants were asked to verbally state how their pain sensation felt at that very instant. The first author (KRT) recorded each response. One question regarding the temporal nature of neuropathic pain was excluded, because it was not meaningful given the acute nature of the present study. Item scores were averaged to form a composite pain score at pre-, post- and 90-min post-exercise. The NPS has been shown to have sensitivity to detect acute treatment effects (30) and has been validated among people with various neuropathic pain syndromes (including SCI).

##### Affect

Hardy and Rejeski's (31) 11-point, single item Feeling Scale (FS) was used to measure participants' overall acute feeling of pleasure-displeasure (−5 = very bad, +5 = very good). In addition, Svebak and Murgatroyd's (32) Felt Arousal Scale (FAS) measured participants' perceived activation (1 = low arousal, 6 = high arousal), whereby “1” indicates feeling bored, relaxed or calm, and “6” indicates feeling excited, angry or frustrated. Using the FS alongside the FAS enhances construct validity by measuring activation in addition to affective valence [pleasure-displeasure; (33)]. Although the FS and FAS have not been validated in the SCI population, previous research has demonstrated the utility of these questionnaires for measuring affect in individuals with SCI participating in acute exercise (9, 10).

#### Protocol

##### Peak Power Output Graded Aerobic Exercise Test

Upon arriving at the lab, participants were given opportunity to use the toilet. After 5 min of seated rest, resting heart rate and blood pressure were measured. KRT explained how to use the Borg Rating of Perceived Exertion [RPE; (34)] (6–20) scale, adapted for persons with SCI (35). Participants then began the peak power output exercise test using a wall-mounted arm ergometer (Lode Angio, Groningen, Netherlands), with the height adjusted to align with the acromioclavicular joint. If required, participants' hands were bound to the handles of the arm ergometer with elastic tensor bandages. The test began with a 5-min warm-up at a self- selected cadence. Using a continuous graded exercise protocol (36), the resistance on the ergometer was adjusted such that power output was increased by 2 W/min for tetraplegics (37) and 10 W/min for paraplegics (38). Participants were asked to maintain a cadence of 55–65 revolutions per min (RPM) until volitional exhaustion. Within the last 10-s of each min of exercise, participants reported their RPE. The maximum wattage achieved during this exercise test was used to calculate the intensity (60% of maximum) of each participant's subsequent sub-maximal intensity aerobic exercise bout (operationalized as 60% peak power output).

##### Sub-maximal Aerobic Exercise Bout

**Figure 1:**
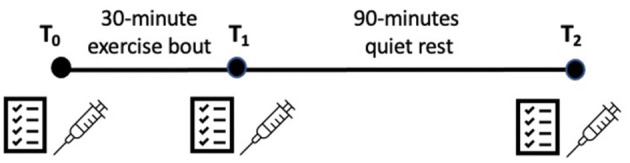
Graphical representation of sub-maximal exercise protocol. Questionnaire symbol = neuropathic pain scale, felt arousal scale and feeling scale; syringe symbol = blood draws.

Prior to arriving at the lab to complete the sub-maximal exercise bout, participants fasted for 12-h. Upon arrival, participants rested quietly for 10 min. Next, participants completed their baseline (T_0_) measurements which consisted of verbally reporting their NPS, FS, and FAS ratings. Blood samples (TNF- α, IL-6, IL-1ra, IL-10) were then collected by the trained phlebotomist. Prior to exercising, KRT reminded participants how to use the “6–20” RPE scale (34, 35). For the exercise bout, participants performed a 5-min warm-up at a self-selected pace and then performed 30-min of arm crank exercise at 60% of their maximum wattage, while maintaining a cadence of 55–65 RPM. Participants were prompted to report their RPE at the end of each minute during this exercise bout. If participants reported their RPE to be above “sub-maximal” (>16), the wattage was electronically lowered to 50% of participants maximum wattage, until their RPE recovered to “12–14,” Immediately following exercise (T_1_), participants verbally reported their NPS, FS, and FAS ratings, and provided another blood sample. Participants were then asked to quietly rest in a room separate from the testing room, and watch a video [“Planet Earth”; (BBC America)] for 90 min. After 90-min (T_2_), participants repeated the NPS, FS, and FAS measures, and their blood was drawn one final time. The order of administration of the NPS, FS, and FAS was systematically randomized at each measurement timepoint to control for presentation biases. See Figure 1 for graphical representation of study protocol.

### Statistical Analyses

One-way repeated measures analysis of variance (ANOVA) with planned contrasts were conducted to assess change in neuropathic pain, cytokines and affect from pre- to post-exercise. Planned contrasts were the appropriate statistical method given that they allow for comparisons of just two means (of a set of means >2). The hypotheses of this study were based on changes in each dependent variable from pre- to post-exercise (i.e., not T_1_T_2_). Therefore, computing planned contrasts allowed for scientifically sensible comparisons and minimized the risk of a Type 1 error. Significance was set at *p* < 0.05. Effect sizes were calculated as Cohen's *d*_av(average)_ with Hedge's g_av_ correction applied (39) and interpreted according to Cohen's conventions [small = 0.20, medium = 0.50, large = 0.80; (40)].

Simple change scores (Δ) were calculated for change in cytokine levels between T_0_T_1_ and T_0_T_2_. To control for the correlation between baseline and subsequent measures of the self-reported variables, residualized change scores were computed to measure change in NPS, FS, and FA scores between T_0_T_1_ and T_0_T_2_. Pearson's correlation coefficients were then computed to determine: (a) the relationships between change in neuropathic pain, change in inflammatory cytokines, and change in affect and arousal at timepoints T_0_T_1_, and T_0_T_2_, and (b) whether baseline levels of inflammatory cytokines were related to change in neuropathic pain at timepoints T_0_T_1_ and T_0_T_2_. Two-tailed tests were used. Consistent with Widerstrom-Noga's recommendations (41), analyses were conducted separately for persons with tetraplegia and paraplegia. Cohen's conventions (40) were used for interpreting the magnitude of the correlations (small = 0.1, medium = 0.3, large = 0.5). SPSS version 22.0 was used for all analyses.

## Results

One male participant with tetraplegia withdrew from the study due to a pressure sore (unrelated to the study protocol). Additionally, the phlebotomist was unable to obtain blood from one male participant; his data are not included in the analyses. Therefore, the following results include data from 8 participants [4 tetraplegics (all male), and 4 paraplegics (3 male, 1 female)]. The average age of participants was 37.9 ± 10.9 years, and ranged between 25 and 56 years. Participants' average years post-SCI was 17.8 ± 8.9, and ranged between 3 and 36 years. Three participants were consuming cannabis at the time of study participation, however, no participants were taking pharmaceuticals at the time of study participation. Participant demographics for the 8 people who completed the study are presented in Table 1.

**Table 1:** Demographic information of the sample (*n* = 8).

**Participant**	**Sex**	**Age**	**Level of injury**	**AIS classification**	**Cause of SCI**
1	M	56	T7	AIS A	Traumatic
2	M	32	T4-T5	AIS B	Traumatic
3	M	36	C6/C7	AIS B	Traumatic
4	F	56	T12-L1	AIS C	Traumatic
5	M	42	C5-C6	AIS C	Traumatic
6	M	35	C6/C7	AIS A	Traumatic
7	M	29	C6/C7	AIS C	Traumatic
8	M	25	T12/L1	AIS B	Traumatic

### Exercise Intensity Manipulation Check

**Figure 2:**
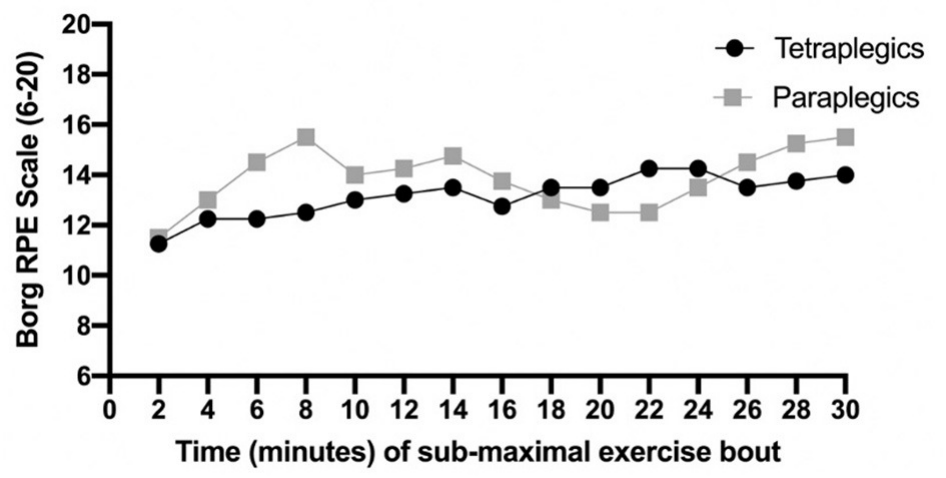
Average of participants' RPE at 2-min intervals throughout the sub-maximal exercise bout.

Manipulation checks were used to verify that participants were exercising at the intended training intensity. Mean_RPE_ during the exercise bout did not significantly differ between tetraplegic and paraplegic participants (M_RPE_ tetra = 13.07 ± 1.10, M_RPE_ para = 13.73 ± 0.69; *t* = −1.02, *p* = 0.35). Similarly, percent change in RPE between T_0_T_1_ did not significantly differ between tetraplegic and paraplegic participants [%_change_ tetra = 47.6 ± 24.49, %_change_ para = 47.48 ± 35.40; *F*_(1, 6)_ = 0.702, *p* = 0.99]. Max_RPE_ (i.e., highest RPE reported during the exercise bout) for tetraplegic participants ranged from 12 to 15, indicating they were all exercising at a “somewhat hard to hard intensity” (32). For the paraplegic participants, Max_RPE_ ranged from 16 to 20, indicating that at certain points, they were exercising at “very hard intensity to exhaustion” (34). Results from an independent samples *t*-test indicated that average Max_RPE_ values for these two groups were significantly different (Tetra: *M* = 14.00 ± 1.4; Para: *M* = 17.25 ± 1.89; *t* = −2.71, *p* = 0.03). These data indicate that, consistent with the goal of the exercise manipulation, tetraplegic participants continuously exercised at a sub-maximal exercise intensity. However, paraplegic participants exceeded a sub-maximal intensity during their exercise bout (see 2-min average RPEs in Figure 2), and at points, may have exercised closer to maximal intensity.

### Direct Effects of Exercise on NPS Scores, Affect, Arousal, and Inflammatory Cytokines

Given the small number of participants, results are reported and interpreted based on the magnitude of the effect sizes. Data are shown in Tables 2–5.

**Table 2:** Changes in the study outcome measures among persons with tetraplegia (*n* = 4).

		**T**_**0**_ **T**_**1**_					**T**_**0**_ **T**_**2**_			
	**Pre-exercise**	**Post-exercise**	***p*-value**	**Effect sizes^**σ**^**	**95% CI**	**Pre-exercise**	**90 min post-exercise**	***p*-value**	**Effect sizes^**σ**^**	**95% CI**
Total NPS	2.60 (1.57)	1.75 (1.01)	0.10	−0.47	(−0.32, 2.02)	2.60 (1.57)	2.75 (1.70)	0.58	0.07	(−0.61, 0.91)
Feeling Scale	2.00 (1.41)	2.5 (0.58)	0.50	0.34	(−1.55, 2.55)	2.00 (1.41)	2.25 (0.50)	0.81	0.17	(−2.75, 3.25)
Felt Arousal Scale	2.50 (0.58)	3.00 (0)	0.18	0.89	(−0.42, 1.42)	2.50 (0.58)	2.50 (0.58)	1.00	——	————–
IL_6_	1.72 (2.83)	1.63 (2.75)	0.26	−0.02	(−0.15, 0.33)	1.72 (2.83)	1.45 (1.59)	0.76	−0.09	(−2.35, 2.88)
IL_10_	0.40 (0.34)	0.33 (0.31)	0.06	−0.16	(−0.00, 0.14)	0.40 (0.34)	0.36 (0.33)	0.11	−0.09	(−0.02, 0.10)
IL_1RA_	137.4 (124.1)	143.1 (125.9)	0.40	0.03	(−14.50, 25.69)	137.4 (124.1)	143.1 (127.40)	0.33	0.03	(0.45, 10.95)
TNF-a	1.62 (1.31)	1.70 (1.45)	0.54	0.04	(−0.30, 0.46)	1.62 (1.31)	1.59 (1.35)	0.57	−0.02	(−0.12, 0.18)

*Data are presented as Mean (SD); ^σ^Hedge's g_av_; Neuropathic Pain Scale (NPS) values are a composite value of 10 items rated on a numerical rating scale ranging from 0 to 10 (0 = no pain, 10 = worst pain imaginable). Feeling Scale (FS) is a single item rated on a bipolar scale (−5 = very bad, +5 = very good). Felt Arousal Scale is a 6-item scale rated on a 1–6 numerical rating scale (1 = low arousal, 6 = high arousal)*.

*Cytokines are measured in pg/ml*.

**Table 3:** Correlations between changes in pain, changes in affect, changes in arousal, and changes in cytokines among persons with tetraplegia (*n* = 4).

**T**_**0**_ **T**_**1**_			**T**_**0**_ **T**_**2**_	
**Correlations**	***r***	***p*-value**	***r***	***p*-value**
*r* Δ NPS/ΔFS	−0.67	0.33	−0.67	0.33
*r* Δ NPS/ΔFAS	−0.75	0.25	−0.94	0.06
*r* Δ NPS/ΔIL6	−0.82	0.18	−0.67	0.33
*r* Δ NPS/ΔIL10	−0.40	0.61	−0.97^*^	0.04
*r* Δ NPS/ΔIL1RA	−0.62	0.38	−0.18	0.83
*r* Δ NPS/ΔTNF-a	0.49	0.52	0.83	0.17
*r* Δ NPS/MaxRPE	−0.91^**^	0.04	−0.68	0.16

* *p < 0.05 (2-tailed)*;

** *p < 0.05 (1-tailed). Bonferroni adjusted for multiple comparisons*.

**Table 4:** Changes in the study outcome measures among persons with paraplegia (*n* = 4).

		**T**_**0**_ **T**_**1**_					**T**_**0**_ **T**_**2**_			
	**Pre-exercise**	**Post-exercise**	***p*-value**	**Effect sizes^**σ**^**	**95% CI**	**Pre-exercise**	**90 min post-exercise**	***p*-value**	**Effect sizes^**σ**^**	**95% CI**
Total NPS	1.83 (1.45)	1.53 (1.39)	0.21	−0.15	(−0.30, 0.90)	1.83 (1.45)	1.28 (1.05)	0.09	−0.32	(−0.16, 1.26)
Feeling Scale	2.75 (1.26)	2.0 (0.82)	0.44	−0.51	(−1.97, 3.47)	2.75 (1.26)	2.75 (1.26)	1.00	—–	————–
Felt Arousal Scale	2.75 (1.26)	3.50 (1.29)	0.32	0.43	(0.28, 1.22)	2.75 (1.26)	2.00 (0.82)	0.06	−0.51	(−0.04, 1.54)
IL_6_	1.40 (0.13)	1.58 (0.35)	0.39	0.66	(-0.39, 0.75)	1.40 (0.13)	1.30 (0.17)	0.42	−0.66	(−0.26, 0.46)
IL_10_	0.25 (0.19)	0.21 (0.16)	0.063	−0.17	(−0.01, 0.09)	0.25 (0.19)	0.23 (0.20)	0.29	−0.07	(−0.04, 0.08)
IL_1RA_	179.7 (90.8)	198.0 (123.9)	0.79	0.12	(−178.6, 215.2)	179.7 (90.8)	161.9 (45.24)	0.53	−0.18	(−61.0, 96.6)
TNF-a	1.15 (0.84)	1.08 (0.81)	0.63	−0.08	(−0.37, 0.51)	1.15 (0.84)	1.11 (0.85)	0.69	−0.03	(−0.28, 0.36)

*Data are presented as Mean (SD); ^σ^Hedge's g_av_; Neuropathic Pain Scale (NPS) values are a composite value of 10 items rated on a numerical rating scale ranging from 0 to 10 (0 = no pain, 10 = worst pain imaginable). Feeling Scale (FS) is a single item rated on a bipolar scale (−5 = very bad, +5 = very good). Felt Arousal Scale is a 6-item scale rated on a 1–6 numerical rating scale (1 = low arousal, 6 = high arousal)*.

*Cytokines are measured in pg/ml*.

**Table 5:** Correlations between changes in pain, changes in affect, changes in arousal, and changes in cytokines among persons with paraplegia (*n* = 4).

**T**_**0**_ **T**_**1**_			**T**_**0**_ **T**_**2**_	
**Correlations**	***r***	***p*-value**	***r***	***p*-value**
*r* Δ NPS/ΔFS	0.22	0.78	−0.18	0.82
*r* Δ NPS/ΔFAS	0.33	0.67	−0.53	0.47
*r* Δ NPS/ΔIL6	−0.60	0.40	0.83	0.17
*r* Δ NPS/ΔIL10	0.79	0.21	0.17	0.83
*r* Δ NPS/ΔIL1RA	−0.40	0.60	0.32	0.68
*r* Δ NPS/ΔTNF-a	0.70	0.30	0.42	0.58
*r*Δ NPS/ MaxRPE	0.63	0.19	−0.83	0.08

*Bonferroni adjusted for multiple comparisons*.

For persons with tetraplegia, between T_0_T_1_ there was some evidence of clinically meaningful changes in neuropathic pain [i.e., reductions > 30%; (42)] and improvements in affect and arousal. Effect sizes for changes in NPS scores, affect, and arousal between T_0_T_1_ were medium-to large (g_av_ = −0.47, 0.34, and 0.89, respectively). Changes in inflammatory cytokines between T_0_T_1_ were very small (g_av_ range: 0.02–0.16). Between T_0_T_2_, effect sizes for changes in NPS scores and affect were very-small to small (0.07 and 0.17, respectively). Effect sizes for arousal did not change between T_0_T_2_. Effect sizes for changes in inflammatory cytokines between T_0_T_2_ were also very-small (*g*_av_ range: 0.02–0.09).

For persons with paraplegia, calculation of the effect sizes provided support for some notable changes in pain, affect, and cytokine measures at both time points. Between T_0_T_1_, the effect size for change in NPS scores was small (−0.15), and medium for affect and arousal (−0.51, 0.43, respectively). Effect sizes for change in inflammatory cytokines between T_0_T_1_ were very-small (0.08–0.17), except for IL-6 which was medium-large (0.66). Between T_0_T_2_, change in NPS scores were small- to medium (−0.32), and change in arousal was medium (−0.51). Effect sizes for affect did not change between T_0_T_2_,. Effect sizes for change in T_0_T_2_ cytokines were very small (0.03–0.18), except for IL-6 which was medium-large (0.66).

### Correlations Between Change in Inflammatory Cytokines and Change in Neuropathic Pain

For persons with tetraplegia, there were medium-to large, negative correlations between change in cytokine levels and change in NPS scores between T_0_T_1_ (*rs* ranged from −0.82 to 0.49), and T_0_T_2_ (*rs* > −0.67), except for IL-1ra (*r* = −0.18; Table 3).

For persons with paraplegia, correlations between change in T_0_T_1_ levels of inflammatory cytokines and change in NPS scores were medium to large (*rs* ranged from −0.60 to 0.79; Table 5) but were not in a consistent direction immediately post-exercise. However, correlations between change in NPS scores and change in levels of cytokines between T_0_T_2_ were small to medium (*r*s ranged from 0.17 to 0.42), except for IL-6 which was large (*r* = 0.83).

### Correlations Between Change in Neuropathic Pain and Change in Affect

For persons with tetraplegia, as hypothesized, there were medium- to large, negative correlations between change in NPS scores, and change in FS and FA scores between T_0_T_1_, (*rs* > −0.67), and T_0_T_2_ (*rs* > −0.67).

For persons with paraplegia, between T_0_T_1_, small- to-medium positive correlations were observed between change in NPS scores and change in FS scores (*r* = 0.22), and FA scores (*r* = 0.33). In contrast, between T_0_T_2_, small-to medium, negative correlations were observed between change in NPS scores and FS scores (*r* = −0.18) and FA scores (*r* = −0.53).

### *Post-hoc* Correlations Between Maximum Ratings of Perceived Exertion and Change in Neuropathic Pain

The hypotheses of the current study were based on acute responses to exercise at a sub-maximal intensity. However, the manipulation check of Max_RPE_ data revealed tetraplegic participants were indeed exercising at a sub-maximal intensity, but paraplegic participants may have surpassed this sub-maximal level at certain points in the exercise bout, and approached an exercise intensity near maximal. As previous research among chronic pain populations indicates that *extremely* high exercise intensities may acutely increase experimentally induced pain (43, 44), a *post-hoc* decision was made to compute Pearson's correlations between Max_RPE_ and NPS scores to determine if the relationship between Max_RPE_ is different for tetraplegic vs. paraplegic participants.

For persons with tetraplegia, there were medium-to-large, negative correlations between Max_RPE_ and change in NPS scores between T_0_T_1_ (*r* = −0.91) and T_0_T_2_ (*r* = −0.68).

In contrast, for persons with paraplegia, a medium-to-large positive correlation was found between Max_RPE_ and change in NPS scores between T_0_T_1_ (*r* = 0.63). However, between T_0_T_2_, a large, negative correlation was observed between Max_RPE_ and change in NPS scores (*r* = −0.83).

### Correlations Between Baseline Levels of Cytokines and Change in Neuropathic Pain

**Figure 3:**
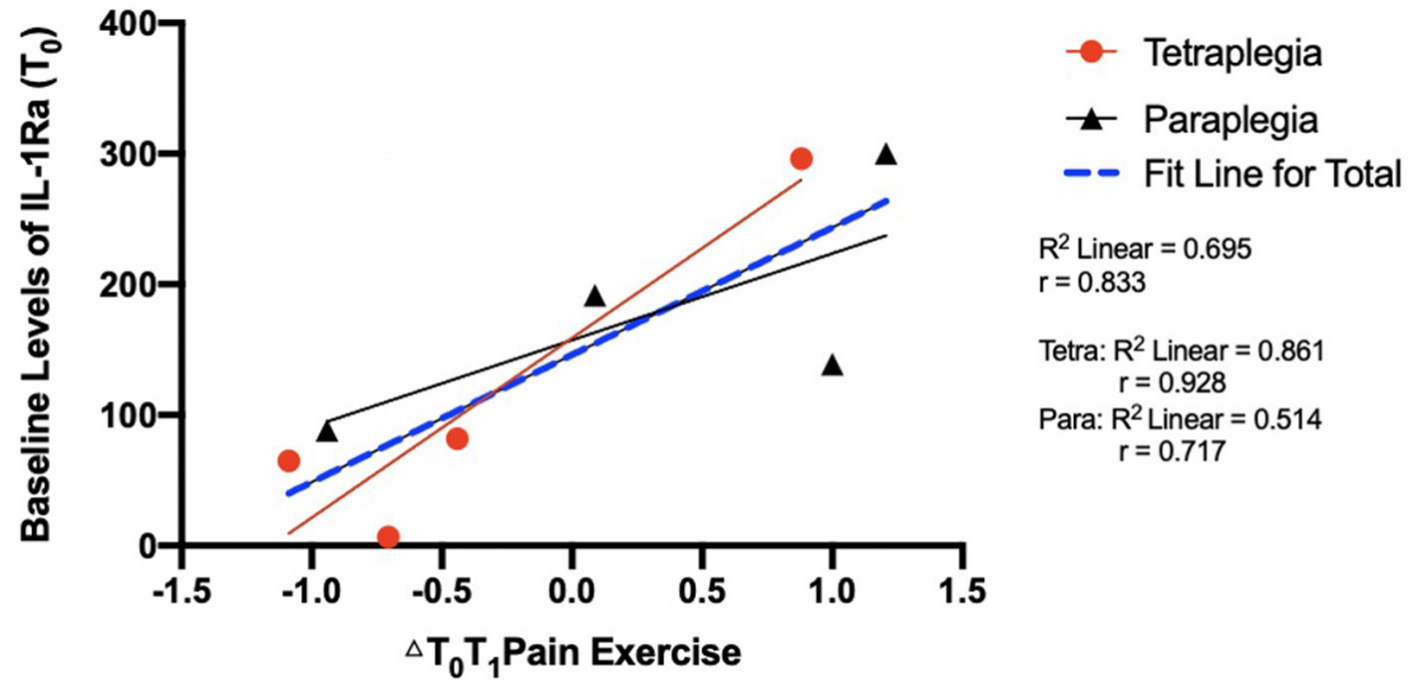
Scatterplot of baseline levels of IL-1Ra and changes in neuropathic pain between T_0_T_1_.

A large-sized significant, positive correlation was observed between baseline levels of IL-1ra and change in NPS scores between T_0_T_1_ (*r* = 0.833; Table 6; Figure 3). However, this relationship was not sustained 90-min post-exercise (*r* = −0.17). Correlations between baseline levels of all other cytokines and change in NPS scores were inconsistent in size and direction at timepoints T_0_T_1_ and T_0_T_2_ (*rs* = −0.27–0.55; Table 6).

**Table 6:** Correlations between baseline levels of cytokines and changes in pain for all study participants (*n* = 8).

**T**_**0**_ **T**_**1**_			**T**_**0**_ **T**_**2**_	
**Correlations**	***r***	***p*-value**	***r***	***p*-value**
*r* baseline IL-6/Δ*NP*	0.55	0.155	0.24	0.576
*r* baseline IL-10/Δ*NP*	−0.27	0.521	0.11	0.793
*r* baseline IL-1ra/Δ*NP*	0.83^**^	0.010	−0.17	0.687
*r* baseline TNF-a/Δ*NP*	0.004	0.993	0.27	0.52

** *p ≤ 0.01 (2-tailed). Bonferroni adjusted for multiple comparisons*.

## Discussion

The primary purpose of this study was to test the acute effects of sub-maximal aerobic exercise on inflammatory cytokines, neuropathic pain, affect and arousal among individuals with SCI. Additionally, this study aimed to explore if the baseline levels of cytokines may be related to exercise-related changes in neuropathic pain. Consistent with our hypotheses, exercise led to decreased levels of neuropathic pain, and improved affect, in both participants with tetraplegia and paraplegia. However, changes in inflammatory cytokines following exercise participation were inconsistent in direction. For example, a small-sized acute decrease in IL-6 was observed for persons with tetraplegia following exercise participation, whereas a medium-large acute increase in IL-6 was observed for persons with paraplegia. Interestingly, lower baseline levels of IL-1Ra were significantly related to greater acute decreases in neuropathic pain. To the best of our knowledge, this is the first SCI study to evaluate the effects of exercise on neuropathic pain, inflammatory cytokines and affect.

The observed positive effects of exercise on neuropathic pain align with previous SCI research (6, 7, 9–11). However, previous research suggests that exercise reduces neuropathic pain to a similar extent for paraplegics and tetraplegics. In our study, these effects were large for tetraplegics and small for paraplegics. These differences may be at least partly attributable to the significant differences in intensity at certain points of the exercise bout, as reported by tetraplegic vs. paraplegic participants.

Indeed, research in able-bodied individuals suggests that the analgesic effects of exercise are elicited once exercise exceeds a threshold of intensity. Moderate- to high-intensity exercise [but not maximal; (14, 45)], has been shown to lead to greater reductions in pain compared to lower intensity exercise in other chronic health populations. However, performing exercise that is perceived to be “very hard to exhaustive” may increase pain, at least in the short-term. Paraplegics reported exercising at a “very hard” subjective intensity at certain points during this exercise bout, and their neuropathic pain and Max_RPE_ were positively correlated immediately post-exercise. The correlation became negative at 90-min post-exercise, paralleling greater reductions in neuropathic pain at 90-min post-exercise for this group. Research suggests that exercise-induced hypoalgesia remains for ≤ 30 min after exercise, but may be impaired in individuals with chronic pain (14). Therefore, paraplegic participants may have highly exerted themselves during this bout of exercise, which inhibited exercise-induced hypoalgesia immediately after exercise completion. Exercising “near maximal” intensity, may have conflated paraplegic participants immediate post-exercise neuropathic pain reports, with muscle soreness. Ninety minutes of recovery may have allowed for sufficient rest, and for paraplegic participants to experience the benefits of high-intensity exercise, such as reduced neuropathic pain. Future research must continue to examine the impact of exercise intensity on neuropathic pain in persons with SCI, given the distinctly different relationships with neuropathic pain when participants met, vs. exceeded a “hard” (34) exercise intensity in this study.

Among the able-bodied population, research evidence indicates that exercise-related changes in IL-6 is intensity dependent (46), and IL-6 can be sizeably increased compared to baseline levels following high-intensity exercise (47). Results from this study support previous findings, given the large increase in IL-6 that occurred from pre- to post-exercise for persons with paraplegia (i.e., “very hard”) exercisers. IL-6 is an inflammation-controlling cytokine and stimulates the exercise-related anti-inflammatory cascade, which suggests that tetraplegic participants may not have been exercising at a high enough intensity to initiate increased levels of anti-inflammatory cytokines. Research evidence also indicates that exercise-related increases in IL-6, and the subsequent anti-inflammatory cascade is further responsible for eliciting analgesic effects (17, 18, 48). However, results from this study do not align with this evidence, given that a stronger correlation was observed between IL-6 and neuropathic pain for persons with tetraplegia. Therefore, conflicting correlations between neuropathic pain and inflammatory cytokines, coupled with the significant difference in Max_RPE_ reported between levels of injury, suggests the inflammatory etiology of exercise-related changes in neuropathic pain among persons with SCI may also be impacted by exercise intensity. Future research should test the mediating effects of inflammation, by identifying whether specific exercise intensities lead to decreases in neuropathic pain, through effects on the inflammatory cascade.

Although impaired autonomic nervous systems may contribute to the conflicting pattern of correlations observed for tetraplegic vs. paraplegic participants between neuropathic pain, affect and arousal, differences in exercise intensity reported between these groups may help further explain these correlations. The intensity of exercise required to stimulate optimal affective responses remains highly debated (43, 44, 49). For individuals with SCI, previous literature demonstrates that acute exercise-related decreases in pain are correlated with improvements in feeling states (9, 10). However, the intensity and type of exercise prescribed within these previous studies were inconsistent. For persons with tetraplegia, the large, negative correlations between neuropathic pain, affect and arousal align with previous research (9, 10). In contrast, for persons with paraplegia, exercise-related changes in neuropathic pain were positively correlated with changes in feeling states immediately post-exercise, and negatively correlated 90 min post-exercise_._ Indeed, moderate vs. high-intensity exercise has been shown to differentially impact affective responses and pain sensations in the general population (43). Future research should be directed toward understanding the impact of exercise intensity on these constructs among persons with SCI.

Further, the timepoint of assessments of affective responses may impact the interpretation of results. For participants with paraplegia, conflicting affective responses immediately post-exercise vs. 90-min post-exercise may be explained by the rebound model (49). High-intensity exercise often stimulates negative affective responses immediately post-exercise, whereas these responses become positive after a period of recovery. Paraplegic participants exercised near maximal intensity and may have experienced a disruption in physiological homeostasis immediately post-exercise. However, 90-min of rest may have been sufficient for paraplegics to recover and experience an affective “rebound.” While the intensity of exercise and timing of affective assessments are not presumed to fully explain participants' affective response to exercise nor its correlation with neuropathic pain, they are presented here as potential exercise protocol characteristics that may partially explain these relationships. Future psychophysiological SCI-exercise research should also investigate the impact of additional social-cognitive and physiological predictors of affective responses to exercise (e.g., exercise self-efficacy, thermoregulation, and ventilatory threshold) and their relationship with neuropathic pain.

In addition to its primary purpose, this study provided the opportunity to assess whether inflammatory cytokines may be used as predictive biomarkers to determine individuals likely to benefit from exercise. Lower levels of IL-1ra at baseline were associated with larger exercise- related reductions in neuropathic pain. IL-1ra is an anti-inflammatory cytokine which is stimulated by post-exercise increases in IL-6. Therefore, individuals who have lower baseline levels of IL-1ra may have a greater capacity for upregulation of IL-1ra in response to exercise, thereby inducing anti-inflammatory and subsequent analgesic effects. Results from this study support previous research among persons with pain (e.g., knee osteoarthritis), whereby levels of plasma IL-1ra have been shown to predict response to treatment (50). Future research, including the RCT associated with this pilot study (51), should continue to investigate IL-1ra as a potential predictive biomarker for exercise-related changes in neuropathic pain among persons with SCI. Better understanding predictive biomarkers will enhance our knowledge of who may experience improvements in neuropathic pain following exercise, and therefore inform patient care decisions (52).

### Study Strengths and Limitations

This study has several strengths. First, collecting multiple measurements and observing possible pathways responsible for exercise-related changes in pain allowed for a deeper evaluation of the relationship between exercise and neuropathic pain among persons with SCI. Results of this study provide rationale to further examine potential mechanisms impacting exercise-related changes in neuropathic pain, such as whether the intensity of exercise leads to decreased neuropathic pain, through its effects on inflammation among individuals with SCI. Second, evaluating the effect of exercise on neuropathic pain among humans with SCI (rather than animals), allowed for use of clinically relevant measurement tools and the ability to measure spontaneous neuropathic pain. Evaluating animal models would have precluded evaluating psychosocial contributors to neuropathic pain, given the difficulty of assessing affective measures of neuropathic pain in pre-clinical research (53). Third, assessing the concomitant impact of exercise on neuropathic pain, inflammation and affect allowed for a greater understanding of the dynamic interaction among physiological (i.e., inflammation) and psychological (i.e., affect) contributors to neuropathic pain.

Despite these strengths, some limitations must be noted. First, participants' individual sub-maximal exercise intensity was based on their peak power output rather than their VO_2peak_. Although we intended for all participants to exercise at 60% peak power output, there was a significant difference in Max_RPE_ reported between tetraplegic and paraplegic participants at certain points, and paraplegic participants likely exceeded a sub-maximal exercise intensity. Further, paraplegic participants approached an “exhaustive” RPE (34) halfway through the exercise bout, which prompted the research team to decrease their wattage from 60 to 50% peak power output for 5 min to allow for a brief recovery period. Despite this limitation, the significantly different Max_RPE_ reported between tetraplegic vs. paraplegic participants provided valuable insight into the potential impact of exercise intensity on neuropathic pain, inflammatory cytokines, affect and arousal among individuals with SCI. Second, although International SCI Pain Data Sets have been introduced to measure neuropathic pain in adults with SCI (54), we employed the NPS due to its brevity, ease of comprehension, and ability to assess responses to treatment (30). Future research should incorporate the International SCI Pain Data Set in order to ensure proper identification of participants' pain, and to allow for comparison of results across studies (51). Third, the FS and FAS have been used extensively in exercise research to investigate individuals' affective responses to exercise. Additional research is needed to assess the validity of the FAS when used in exercise contexts with persons with SCI. Fourth, this study included a small number of participants, and only one female, which is limiting due to the research evidence supporting sex differences in post-exercise immune and pain responses (55, 56). While the ratio of male to female participants in this study is representative of the global SCI population (57), future SCI research must strengthen recruitment strategies to include more female participants and enhance the generalizability of findings. The small sample size may have influenced our results (in terms of both effect size and lack of statistical significance), and these results should be interpreted with caution as they were not all statistically significant. Fifth, this study evaluated only inflammation as a possible pathway responsible for exercise-related changes in neuropathic pain. It is understood that many additional mechanisms (e.g., microglial activation, cortisol levels, pain catastrophizing) may be responsible for exercise-related changes in neuropathic pain among adults with SCI. To progress toward mechanism-based treatment, future SCI research should investigate further mechanisms that may impact the relationship between exercise and neuropathic pain. And finally, we did not employ a control condition against which to evaluate the effects of the exercise bout. Designing a true control for tests of pain-reducing interventions is challenging, but should be considered in future studies to control for the effects of attention, distraction and other psychological variables on neuropathic pain and affect.

### Conclusion

Taken together, the results of this study suggest that exercise may reduce neuropathic pain and improve affect in adults with SCI, and changes in inflammation may be related to these effects. Additionally, exercise intensity may play an important role in the exercise related changes in neuropathic pain, inflammatory profiles, and affect for adults with SCI. Last, levels of IL-1ra may help determine who experiences exercise-related reductions in neuropathic pain sensations. Future research should be directed toward understanding the ideal exercise intensity for decreasing neuropathic pain among adults with SCI, and the potential role of inflammatory cytokines and other possible mediators.

## Data Availability Statement

The raw data supporting the conclusions of this article will be made available by the authors, upon reasonable request.

## Ethics Statement

The studies involving human participants were reviewed and approved by University of British Columbia Clinical Research Ethics Board. The patients/participants provided their written informed consent to participate in this study.

## Author Contributions

KT was responsible for designing the study protocol, writing the protocol and report, participant recruitment, data collection, analyzing data, interpreting results, writing manuscript, updating reference lists, and creating tables and figures. JV, JL, and JK were responsible for designing the study protocol, interpreting results, and manuscript revision. JW was responsible for designing the study protocol, assisting with data collection, interpreting results, and manuscript revision. GJ was responsible for designing the study protocol, analyzing data, and manuscript revision. GD was responsible for designing the study protocol, assisting with data collection and analysis, and revising the manuscript. KMG was responsible for designing the protocol, assisting with writing the protocol and report, analyzing data, interpreting results, manuscript revision, and designing tables and figures. All authors contributed to the conception and design of this study, in addition to drafting, or critically revising the article.

## Funding

This work was supported by the Rick Hansen Foundation through the Blusson Integrated Cures Partnership (2019).

## Conflict of Interest

The authors declare that the research was conducted in the absence of any commercial or financial relationships that could be construed as a potential conflict of interest.

## Publisher's Note

All claims expressed in this article are solely those of the authors and do not necessarily represent those of their affiliated organizations, or those of the publisher, the editors and the reviewers. Any product that may be evaluated in this article, or claim that may be made by its manufacturer, is not guaranteed or endorsed by the publisher.
