# Supplementary material for: The Impact of Sub-maximal Exercise on Neuropathic Pain, Inflammation, and Affect Among Adults With Spinal Cord Injury: A Pilot Study
Source: Front Rehabil Sci. 2021 Oct 26;2:700780. doi: 10.3389/fresc.2021.700780 (PMC9397724; doi:10.3389/fresc.2021.700780)
Supplement: Supplementary file 1 [file Table_1.docx]

|  |  | | ***T_0_T_1_*** | | | | | |  | | ***T_0_T_2_*** | | | | |
| --- | --- | --- | --- | --- | --- | --- | --- | --- | --- | --- | --- | --- | --- | --- | --- |
|  | *Pre- exercise* | | *Post- exercise* | | *p-value* | *Effect Sizes (Hedge’s G_av_)* | | *95% CI* | *Pre-exercise* | *90 min. post-exercise* | | | *p-value* | *Effect Sizes*  *(Hedge’s G_av_)* | *95% CI* |
| Total NPS | 2.21 (1.46) | | 1.64 (1.14) | | 0.033* | -0.39 | | (-0.97,2.12) | 2.21 (1.46) | | | 2.01 (1.53) | 0.353 | -0.12 | (-1.57,1.97) |
| Feeling Scale | 2.38 (1.30) | | 2.25 (0.71) | | 0.826 | -0.11 | | (-1.11,1.37) | 2.38 (1.30) | | | 2.50 (0.93) | 0.802 | 0.09 | (-1.22,1.46) |
| Felt Arousal Scale | 2.63 (0.92) | | 3.25 (0.89) | | 0.095 | 0.61 | | (-0.45,1.69) | 2.63 (0.92) | | | 2.25 (0.71) | 0.197 | -0.41 | (-0.59,1.35) |
| IL_6_ | 1.56 (1.86) | | 1.61 (1.82) | | 0.674 | 0.03 | | (-2.13,2.23) | 1.56 (1.86) | | | 1.37 (1.05) | 0.638 | -0.11 | (-1.60,1.98) |
| IL_10_ | 0.33 (0.27) | | 0.27 (0.24) | | 0.004* | -0.23 | | (-0.24,0.36) | 0.33 (0.27) | | | 0.29 (0.26) | 0.038* | -0.13 | (-0.27,0.35) |
| IL_1RA_ | 146.1 (108.2) | | 170.55(119.29) | | 0.472 | 0.19 | | (-110.19,  159.09) | 146.1 (108.2) | | | 152.54(89.1) | 0.744 | 0.06 | (-110.7,  123.6) |
| TNF-a | 1.39 (1.05) | | 1.39 (1.14) | | 0.959 | ------ | | (-1.29,1.29) | 1.39 (1.05) | | | 1.34 (1.08) | 0.487 | -0.04 | (-1.21,1.30) |
| Max_RPE_ |  |  | |  | | | 15.63 (2.33) | | | | | |  |  |  |
| Mean_RPE_ |  |  | |  | | | 13.4 (0.92) | | | | | |  |  |  |
| %Change_RPE_ |  | |  | | 47.54 (28.19) | | | | | | | | | | |

**Supplementary File (Table 7).** Outcome measures for the study variables for the total study sample (n=8)

Note: Data are presented as Mean (SD); * = p<0.05 (2-tailed); Neuropathic Pain Scale (NPS) values are a composite score of a 0-10 NRS (0 = no pain, 10 = worst pain imaginable). Feeling Scale (FS) is a bipolar scale (-5 = very bad, +5 = very good). Felt Arousal Scale is a 1-6 NRS (1 = low arousal, 6 = high arousal).
